# Value of serum NT proBNP, HMGB1, and SIRT1 in the diagnosis and prognosis of neonatal respiratory distress syndrome

**DOI:** 10.12669/pjms.41.4.10095

**Published:** 2025-04

**Authors:** Qingqing Yang, Hongbin Zhu

**Affiliations:** 1 Qingqing Yang Department of Paediatrics, Maternity & Child Care Center of Qinhuangdao, Qinhuangdao 066000, Hebei, China; 2 Hongbin Zhu Department of Paediatrics, Maternity & Child Care Center of Qinhuangdao, Qinhuangdao 066000, Hebei, China

**Keywords:** HMGB1, NRDS, NT-proBNP, SIRT1

## Abstract

**Objective:**

To analyze the value of serum aminoterminal pro-brain natriuretic peptide (NT-proBNP), High mobility group protein B1(HMGB1), and silent information regulator factor 2 related enzyme 1 (SIRT1) in the diagnosis and prognosis of neonatal respiratory distress syndrome (NRDS).

**Methods:**

This was a retrospective study. Eighty cases of NRDS infants were selected in Maternity & Child Care Center of Qinhuangdao from January 2022 to January 2024 as the study group, with 80 cases of normal newborns during the same period selected as the control group. The NRDS infants were divided into the excellent prognosis group (n=49) and the poor prognosis group (n=31). The levels of serum NT-proBNP, HMGB1, and SIRT1 were compared among the groups, along with an investigation of the value of these biomarkers for the diagnosis and prognosis of NRDS infants.

**Results:**

The study group exhibited higher levels of serum NT-proBNP and HMGB1(P< 0.05) and lower levels of serum SIRT1(P< 0.05) than the control group. The ROC curve analysis suggested that AUC values for serum NT-proBNP, HMGB1, SIRT1, and their combination in the diagnosis of NRDS were 0.903, 0.829, 0.794, and 0.958, respectively. The ROC curve analysis showed that AUC values for serum NT-proBNP, HMGB1, SIRT1, and their combination in predicting poor prognosis in NRDS infants were 0.810, 0.813, 0.741, and 0.935, respectively.

**Conclusion:**

The combination of serum NT-proBNP, HMGB1, and SIRT1 may demonstrate a high diagnostic value for NRDS infants, as well as a high predictive value for poor prognosis in such infants.

## INTRODUCTION

Neonatal respiratory distress syndrome (NRDS) is a severe disease commonly seen in premature infants, primarily caused by a deficiency of pulmonary surfactant (PS), leading to alveolar collapse and progressive dyspnea.^1^-^3^ Due to the rapid onset and progression of NRDS, prompt and accurate diagnosis and prognosis assessment are crucial for decision-making in clinical practice.^4^,^5^ In recent years, with the advancement of molecular biology and biomarker research, the application of serum aminoterminal pro-brain natriuretic peptide (NT-proBNP), high mobility group protein B1 (HMGB1), and silent information regulatory factor 2 related enzyme 1 (SIRT1) as biomarkers in NRDS has gradually gained attention.

Specifically, NT-proBNP, as a marker of cardiac stress and myocardial function impairment, may reflect changes in pulmonary circulation pressure and cardiac dysfunction in NRDS.^6^,^7^ Meanwhile, HMGB1, as an inflammatory mediator, is involved in the inflammatory processes of various diseases. In NRDS, HMGB1 may be involved in lung inflammation reactions and is correlated with the severity and prognosis of the disease.^8^ Additionally, SIRT1 is a critical deacetylase enzyme involved in various biological processes such as cellular metabolism, autophagy, and apoptosis.^9^ In NRDS, SIRT1 may be involved in lung tissue impairment repair and disease progression by regulating pathways such as cellular autophagy and apoptosis. However, there is currently limited research on the above markers in the diagnosis and prognosis of NRDS. Therefore, this study was designed to explore the value of serum NT-proBNP, HMGB1, and SIRT1 in the diagnosis and prognosis assessment of NRDS.

## METHODS

This was a retrospective study. Eighty NRDS infants were selected in Maternity & Child Care Center of Qinhuangdao from January 2022 to January 2024 as the study group, while 80 normal newborns during the same period were selected as the control group.

### Ethical Approval:

The study was approved by the Institutional Ethics Committee of Maternity & Child Care Center of Qinhuangdao(No.:QHDFY-2023041910; date:April 19, 2023).

### Inclusion criteria:

- Infants who met the diagnostic criteria for NRDS according to the “Montreux Criteria for Neonatal Acute Respiratory Distress Syndrome”(2017 edition).^10^
- Chest X-ray showed diffuse negativity in both lungs, characterized by granular ground-glass opacity and reduced transparency, without signs of atrial hypertension.
- Infants who experienced progressive respiratory distress with inspiratory groaning shortly after birth, accompanied by cyanotic three concave signs.
- Parents signed informed consent forms at the hospital.

### Exclusion criteria:

- Infant with severe extrapulmonary infections.
- Infants with congenital heart disease.
- Infants with genetic metabolic disorders.
- Infants with severe congenital organ or systemic malformations.

Peripheral venous blood (three ml) was drawn from all study subjects and centrifuged at 3,500 r/min for 10 minutes to separate serum and stored at -80°C until testing. Serum NT-proBNP, HMGB1, and SIRT1 were measured by enzyme-linked immunosorbent assay using a Varioskan LUX automatic microplate reader (Thermo Fisher Scientific, USA).

### Outcome Indicators:

The infants were divided into the poor prognosis group(n=31) and the excellent prognosis group(n=49) according to whether they were discharged after recovery or discontinued treatment, were transferred to other hospitals, and died due to critical conditions.

### Statistical Analysis:

SPSS 22.0 software was used for data processing. T-test was used for measurement data conforming to normal distribution, the single sample T test should be used in this study, and χ^2^ test was used for counting data expressed as percentages; logistic model analysis was used for multi-factor, and ROC curve was used to analyse the predictive value, α=0.05, and when P<0.05, the difference was statistically significant.

## RESULTS

**Fig.1:**
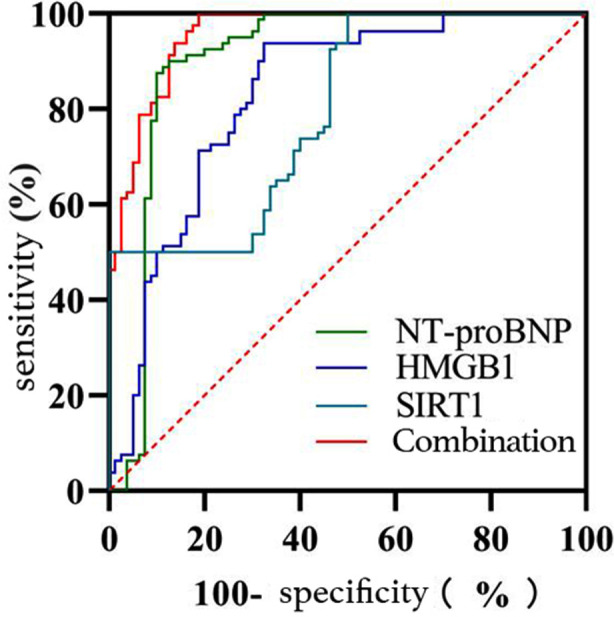
ROC curve analysis of serum NT-proBNP, HMGB1, and SIRT1 in the diagnosis of NRDS.

Baseline data comparison between the two groups showed no statistically significant differences (P >0.05), as shown in Table-I. Compared with the control group, the study group exhibited higher serum NT-proBNP and HMGB1 levels(P<0.05) and lower SIRT1 levels (P < 0.05). Table-II. The levels of serum NT-proBNP, HMGB1, and SIRT1 were included in logistic regression analysis by constructing groups as dependent variables and NT-proBNP, HMGB1, and SIRT1 as independent variables, and the results are shown in Table-III. The combination in the diagnosis of NRDS = NT-proBNP + (0.010/0.005)×HMGB1+ (-0.006/0.005)×SIRT1, and the ROC curve analysis showed that the AUC value of serum NT-proBNP, HMGB1, SIRT1, and their combination in the diagnosis of NRDS was 0.903, 0.829, 0.794, and 0.958, respectively, with the corresponding sensitivities at the optimal cut-off value of 87.5%, 67.5%, 50.0%, and 81.3% and the specificities of 90.0%, 93.7%, 100.0% and 100.0%, respectively. Table-IV and Fig.1. Compared with the excellent prognosis group, the serum NT-proBNP and HMGB1 levels were higher (P <0.05) and the serum SIRT1 levels were lower in the poor prognosis group (P< 0.05), as shown in Table-II.

**Table-I:** Baseline data comparison between the 2 groups (*χ̅*±*S*; *n*,%).

Group	n	Gestational age (weeks)	Gender		Birth weight(kg)		Mode of delivery
			M	F		Spontaneous delivery	Cesarean section
Study	80	35.44±2.43	46 (57.50)	34 (42.50)	3.06±0.83	55 (68.75)	25 (31.25)
Control	80	36.03±1.25	38 (47.50)	42 (52.50)	3.15±0.55	46 (57.50)	34 (42.50)
*t/χ^2^*		-1.923	1.604		-0.813		2.175
*P*		0.056	0.205		0.417		0.140

**Table-II:** Comparison of serum NT-proBNP, HMGB1, and SIRT1 between the two groups, and in NRDS infants with different prognoses (*χ̅*±*S*).

Group	n	NT-proBNP(ng/L)	HMGB1(pg/mL)	SIRT1(ng/L)
Study	80	3852.66±974.47	837.50±154.07	821.65±289.47
Control	80	2893.28±148.16	675.86±80.91	1281.35±425.43
*t*		8.706	8.308	-7.991
*P*		<0.001	<0.001	<0.001
Poor prognosis	31	5243.25±1137.85	942.24±162.15	661.73±213.20
Excellent prognosis	49	3415.76±500.80	771.23±105.19	922.81±287.29
*t*		6.083	5.728	-4.354
*P*		<0.001	<0.001	<0.001

**Table-III:** Results of binary logistic regression analysis.

Influencing factor		B	S.E.	Wald	Significance	EXP(B)	95% C.I. for EXP(B)	
						Lower limits	Upper limits
In study and control groups	NT-proBNP	0.005	0.001	13.511	<0.001	1.005	1.002	1.008
	HMGB1	0.010	0.004	5.325	0.021	1.010	1.002	1.019
	SIRT1	-0.006	0.001	17.057	<0.001	0.994	0.991	0.997
	Constant	-17.981	4.079	19.428	<0.001	0.000		
In NRDS infants with different prognoses	NT-proBNP	0.002	0.001	4.673	0.031	1.002	1.000	1.003
	HMGB1	0.010	0.004	5.146	0.023	1.010	1.001	1.019
	SIRT1	-0.008	0.002	10.922	0.001	0.992	0.987	0.997
	Constant	-9.152	3.274	7.814	0.005	0.000		

**Table-IV:** ROC curve analysis of serum NT-proBNP, HMGB1 and SIRT1 in the diagnosis of NRDS.

Indicator	AUC	SE	Asymptotic significance level	95% CI		Optimal cut-off	Youden index	Sensitivity(%)	Specificity (%)
				Lower limits	Upper limits				
NT-proBNP	0.903	0.029	<0.001	0.846	0.960	3055.13	0.775	87.5	90.0
HMGB1	0.829	0.033	<0.001	0.763	0.894	762.63	0.612	67.5	93.7
SIRT1	0.794	0.035	<0.001	0.726	0.862	761.66	0.500	50.0	100.0
Combination	0.958	0.014	<0.001	0.931	0.986		0.813	81.3	100.0

**Fig.2:**
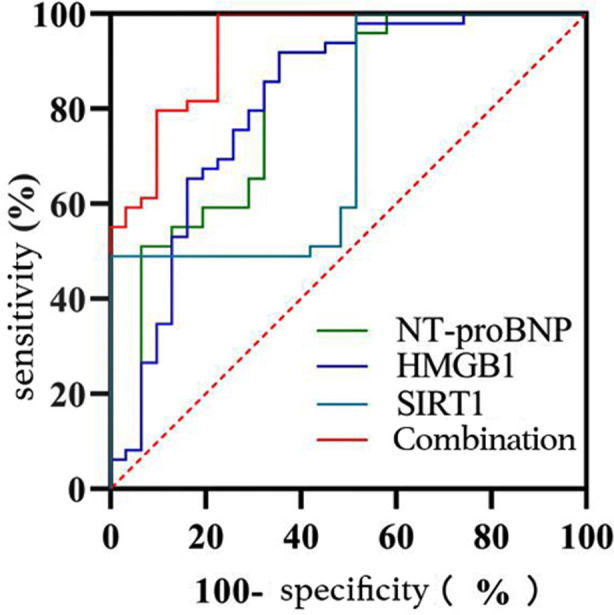
ROC curve analysis of serum NT-proBNP, HMGB1, and SIRT1 in predicting poor prognosisin NRDS infants.

Serum NT-proBNP, HMGB1, and SIRT1 levels were included in logistic regression analysis by constructing groups as dependent variables and NT-proBNP, HMGB1, and SIRT1 as independent variables, and the results are shown in Table-III. The combination in predicting poor prognosis in NRDS infants = NT-proBNP+ (0.010/0.002)×HMGB1+ (-0.008/0.002)×SIRT1. ROC curve analysis showed that the AUC values of serum NT-proBNP, HMGB1, SIRT1, and their combination in predicting poor prognosis in NRDS infants were 0.810, 0.813, 0.741 and 0.935, respectively, with the corresponding sensitivities at the optimal cut-off value of 64.5%, 64.5%, 100.0% and 77.4% and the specificities of 91.8%, 91.8%, 49.0% and 100.0%, respectively. Table-V and Fig.2.

**Table-V:** ROC curve analysis of serum NT-proBNP, HMGB1, and SIRT1 in predicting poor prognosis in NRDS infants.

Indicator	AUC	SE	Asymptotic significance level	95% CI		Optimal cut-off	Youden index	Sensitivity(%)	Specificity(%)
				Lower limits	Upper limits				
NT-proBNP	0.810	0.053	<0.001	0.706	0.913	3935.28	0.563	64.5	91.8
HMGB1	0.813	0.054	<0.001	0.708	0.918	875.965	0.563	64.5	91.8
SIRT1	0.741	0.058	<0.001	0.627	0.855	1017.03	0.490	100.0	49.0
Combination	0.935	0.027	<0.001	0.882	0.988		0.774	77.4	100.0

## DISCUSSION

Serum NT-proBNP, HMGB1, and SIRT1 play significant roles in NRDS infants. The findings of this study showed higher serum NT-proBNP and HMGB1 levels and lower serum SIRT1 levels in the study group than in the study group, which is consistent with the conclusion by Fu X et al.^11^, possibly due to NT-proBNP, as a cardiac stress marker, often being associated with heart function and pressure load. In NRDS, the heart may work harder to exchange oxygen and nutrients to meet the body’s needs due to compromised lung function, which may lead to an increased workload on the heart, thus resulting in elevated NT-proBNP levels.^12^ HMGB1, on the other hand, is an inflammatory mediator typically released during inflammation and infection processes. In NRDS, lung inflammation is a significant pathological process and may cause lung tissue damage and cell death, releasing HMGB1 into the bloodstream and resulting in elevated serum HMGB1 levels.^13^ Moreover, SIRT1 is a silent information regulator factor-related enzyme with anti-inflammatory and antioxidant effects. In NRDS, the activity of SIRT1 may be inhibited due to increased lung inflammation and oxidative stress, leading to a decrease in serum levels.^14^ Meanwhile, the reduced SIRT1 may further exacerbate the processes of inflammation and oxidative stress, forming a vicious cycle. Therefore, the above changes may further aggravate the progression of the disease, which requires prompt medical intervention to improve the prognosis of affected infants.

NRDS, a severe respiratory disease that typically occurs in newborns, especially premature infants, is caused by a deficiency of pulmonary surfactant, which leads to the inability of the lungs to maintain normal expansion during expiration, resulting in dyspnea.^15^ Clinically, NRDS infants often exhibit symptoms such as breathlessness, nasal flaring, and cyanotic skin. As the disease aggravates, affected infants may experience dyspnea, apnea, respiratory failure, and even life-threatening situations.^16^ Therefore, prompt diagnosis and treatment are essential for newborns with NRDS to prevent further aggravation of the condition.

Meanwhile, ROC curve analysis revealed that the AUC values of serum NT-proBNP, HMGB1, SIRT1, and their combination in the diagnosis of NRDS were 0.903, 0.829, 0.794, and 0.958, respectively, with the corresponding sensitivities at the optimal cut-off value of 87.5%, 67.5%, 50.0%, and 81.3% and specificities of 90.0%, 93.7%, 100.0%, and 100.0%, respectively, indicating a high diagnostic value of the combination of serum NT-proBNP, HMGB1, and SIRT1 for NRDS infants. The results of Wang WX et al.^17^ show that the area under ROC curve (AUC) of HMGB1 serum level to predict NRDS is 0.846 (95%CI: 0.755 ~ 0.936). In addition, Xue F et al.^18^ found that the area under the curve (AUC) value of serum HMGB1 level to predict NRDS was 0.872. These conclusions support the results of our study. It is worth emphasizing that the conclusion of this study shows that the combined detection of NT-proBNP, HMGB1, and SIRT1 can offer a more comprehensive understanding of the condition of NRDS infants and assess their cardiac function, pulmonary inflammation, apoptosis, etc., making it a highly valued diagnostic approach. In the meantime, it is also beneficial for guiding clinical treatment and prognosis assessment, thereby providing robust support for the early diagnosis and treatment of NRDS infants.

Additionally, this study also revealed higher serum NT-proBNP and HMGB1 levels and lower serum SIRT1 levels in the poor prognosis group than in the excellent prognosis group, due to the increase of NT-proBNP, a sensitive indicator of cardiac function, being usually associated with increased cardiac burden and cardiac insufficiency. NRDS infants with poor prognosis may experience more severe compromised cardiopulmonary function, leading to increased cardiac pressure, which ultimately causes elevated NT-proBNP levels. In the meantime, the elevated NT-proBNP levels may also reflect the aggravation of systemic inflammatory response and oxidative stress in affected infants, which is also a critical factor in poor prognosis. Additionally, HMGB1 is an essential inflammatory mediator involved in the pathogenesis of a variety of inflammatory diseases, and NRDS infants with poor prognoses may suffer a more intense inflammatory response, resulting in an increased release of HMGB1, while elevated HMGB1 levels may further exacerbate pulmonary inflammation and tissue damage, which forms a vicious cycle, thereby aggravating the condition of the affected infants and affecting their prognosis.^19^ Moreover, SIRT1, an enzyme with anti-aging and anti-inflammatory effects, is essential for maintaining cell homeostasis and reducing tissue damage. NRDS infants with excellent prognoses may exhibit higher SIRT1 levels, which facilitate in reducing the inflammatory response, protecting lung tissue, and improving prognosis. By contrast, decreased SIRT1 levels may indicate diminished anti-oxidative stress and anti-inflammatory capacity in the affected infants, making them unable to effectively respond to NRDS-induced damage, which ultimately leads to their poor prognosis.

On the other hand, changes in these biomarkers are beneficial for assessing the condition and prognosis of NRDS infants and provide a reference for the development of more rational treatment options. Additionally, further ROC curve analysis in this study suggested that the AUC values of serum NT-proBNP, HMGB1, SIRT1, and their combination in predicting poor prognosis in NRDS infants were 0.810, 0.813, 0.741, and 0.935, respectively, with the corresponding sensitivities at the optimal cut-off value of 64.5%, 64.5%, 100.0%, and 77.4% and the specificities of 91.8%, 91.8%, 49.0%, and 100.0%, demonstrating a high predictive value of the combination of serum NT-proBNP, HMGB1, and SIRT1 for poor prognosis in NRDS infants, which was similar to the findings from Wu X.^20^ Given this, the combined detection can reflect the pathophysiological status of NRDS infants in a more comprehensive manner, thereby providing a more accurate prediction for their poor prognosis. Previous studies have mostly explained the diagnostic or predictive value of NT-proBNP, HMGB1 or SIRT1 in NRDS respectively, but this study innovatively explored the diagnostic value and prognosis evaluation value of the combination of the above indicators in children with NRDS.

### Limitations:

This study was a retrospective descriptive study, with limited clinical data available and limited persuasive conclusions. In view of this, further improvements will be made in future research to make more scientific research results.

## CONCLUSIONS

The combination of serum NT-proBNP, HMGB1, and SIRT1 may demonstrate a high diagnostic value for NRDS infants, as well as a high predictive value for poor prognosis in such infants.

### Authors’ Contributions:

**QY:** Carried out the studies, collecting data, and drafted the manuscript, and are responsible and is accountable for the accuracy or integrity of the work.

**HZ:** Performed the statistical analysis and participated in its design, critical analysis.

All authors read and approved the final manuscript.
